# Accrual and drop out in a primary prevention randomised controlled trial: qualitative study

**DOI:** 10.1186/1745-6215-12-7

**Published:** 2011-01-11

**Authors:** Helen C Eborall, Marlene CW Stewart, Sarah Cunningham-Burley, Jackie F Price, F Gerry R Fowkes

**Affiliations:** 1 Department of Health Sciences, University of Leicester, Adrian Building, University Road, Leicester LE1 7RH, UK; 2 Centre for Population Health Sciences, University of Edinburgh, Medical School, Teviot Place, Edinburgh EH8 9AG, UK; 3 Centre for Research on Families and Relationships, University of Edinburgh, 23 Buccleuch Place, Edinburgh EH8 9LN, UK

## Abstract

**Background:**

Recruitment and retention of participants are critical to the success of a randomised controlled trial. Gaining the views of potential trial participants who decline to enter a trial and of trial participants who stop the trial treatment is important and can help to improve study processes. Limited research on these issues has been conducted on healthy individuals recruited for prevention trials in the community.

**Methods:**

Semi-structured interviews with people who were eligible but had declined to participate in the Aspirin for Asymptomatic Atherosclerosis (AAA) trial (N = 11), and AAA trial participants who had stopped taking the trial medication (N = 11). A focus group with further participants who had stopped taking the trial medication (N = 6). (Total participants N = 28).

**Results:**

Explanations for declining to participate could be divided into two groups: the first group were characterised by a lack of necessity to participate and a tendency to prioritise other largely mundane problems. The second group's concern was with a high level of perceived risk from participating.

Explanations for stopping trial medication fell into four categories: side effects attributed to the trial medication; starting on aspirin or medication contraindicating to aspirin; experiencing an outcome event, and changing one's mind.

**Conclusions:**

These results indicate that when planning trials (especially in preventive medicine) particular attention should be given to designing appropriate recruitment materials and processes that fully inform potential recruits of the risks and benefits of participation.

**Trial registration:**

ISRCTN66587262

## Background

Recruitment and retention of participants are critical to the success of a randomised controlled trial (RCT). The CONSORT requirement for RCTs to include a flow diagram of participant passage through the trial has improved the accessibility and scrutiny of accrual and drop-out rates [1,2]. However, reasons for non-participation are not always recorded, investigated or published. Gaining the views of participants (and potential participants) themselves is important for a number of reasons. A growing literature has identified problems with participants making sense of the RCT procedure and the rationale behind randomisation [3-6]; reluctance to accept the principle of equipoise [6]; and preferences for certain treatment arms [5-7]. Indeed when given the choice between being randomly allocated to treatment and receiving one's treatment of choice (in a patient preference trial) randomisation has been found to be unpopular [8]. Investigating trial candidates' views of the recruitment process can help to improve study processes (such as content and delivery of participant information) and recruitment rates [9].

Few studies have accessed in depth the views of individuals who have declined to participate in a trial, though the available evidence suggests that declining is often the result of candidates being unwilling, because of treatment preference, to accept the uncertainty, randomisation and risks associated with an RCT [3,5,7,10]. However, research has mainly focused on patients taking part in trials with overt differences between treatment arms; there is limited research on healthy individuals taking part in research [11,12]; and a particular gap concerning trials of preventive interventions and double-blind placebo controlled trials. Further, the views of trial participants who drop out before the trial completion date have been relatively neglected.

The Aspirin for Asymptomatic Atherosclerosis trial (hereafter referred to as the AAA trial), was a double-blind placebo-controlled randomised trial assessing the effectiveness of low dose aspirin (100 mg/daily) in preventing cardiovascular events in otherwise healthy individuals with asymptomatic atherosclerosis identified by a low ankle brachial index [13]. The index is an effective and simple method of identifying peripheral atherosclerosis [14]; an index of <0.95 is associated with an increased risk of cardiovascular events and mortality [15]. In this paper, we report a qualitative study of people's explanations for declining to participate in the trial (thus affecting accrual rates) or, having begun the trial, stopping the trial medication (thus affecting drop-out rates).

## Methods

A detailed description of the AAA trial procedure has been published [13]. 165,795 individuals identified through general practice records as aged between 50 and 75 years old (on 1^st ^January 1998) were invited to attend for screening with the ankle brachial index. Eligibility criteria for attending included not currently taking aspirin or warfarin and no history of coronary heart disease, stroke or peripheral arterial disease. Of the 29,147 individuals who attended screening, 4914 had an ankle brachial index ≤0.95 (the high risk cut off) in at least one leg, of whom 641 did not meet additional eligibility criteria and 923 declined, leaving 3350 people who were randomised to receive either aspirin (100 mg daily) or placebo for a mean duration of 8.2 (1.6) years [13]. This represents an accrual rate of approximately 8 out of 10 eligible candidates. Non-adherence with study medication throughout the trial was 40% (person-years of follow-up) [13]; 15% of participants took their medication for less than 6 months.

### Qualitative study

Ethical approval was granted by Greater Glasgow (Community/Primary Care), Lanarkshire, and Lothian Local Research Ethics Committees. Individual interviews (N = 22) were conducted with individuals who had attended the screening, were eligible to enter the trial but had declined to participate in the trial (N = 11), and with participants who started the trial but stopped taking the medication (N = 11). A focus group (N = 6) was also conducted with additional trial participants who had stopped the trial medication but who were still receiving annual follow up by the trial team.

### Recruitment

Purposive sampling was undertaken in order to reach individuals with particular relationships to the AAA trial, i.e. people who had declined to participate and trial participants who had stopped the medication, but also to ensure inclusion of people with a range of different lengths of time taking the medication; sampling continued until theoretical saturation was achieved [16].

All eligible individuals who declined to participate in the trial during a 3 month period were sent a participant information leaflet for the qualitative study and opt-in reply slip by post. All those expressing an interest in the qualitative study were telephoned by HCE to discuss participation (including the options to participate in an individual interview or a focus group) and, where willing, to arrange an interview. The accrual rate in this group was approximately 1 out of 10 individuals who were sent an invitation. (NB It was not feasible to arrange a focus group for individuals in the 'declined trial' group due to their widespread locations).

All participants who had stopped trial medication and were scheduled to have an annual phone follow-up within a 1 month period were informed about the qualitative study by the research nurse during the follow-up phone call. As above, all those expressing an interest were telephoned by HCE to check willingness and arrange an interview or focus group. The accrual rate in this group was approximately 1 out of 5 individuals approached.

HCE, a non-clinical social researcher, conducted the semi-structured interviews and focus group using a topic guide based on three main topic areas: the screening, preventive medicine and aspirin, and trial participation. This paper focuses on data generated about the latter area [see appendix for topic guide]. With written consent all interviews and focus groups were audio recorded, transcribed verbatim and anonymised. The data extracts presented in the results section are labelled with the participant's age, gender and group; a number replaces name and location to preserve anonymity.

Analysis was informed by grounded theory and the constant comparative method [17,18]: Transcripts were read and re-read, emerging themes were noted and preliminary (open) codes were formed (by HCE), which further developed throughout the fieldwork. Codes were discussed with SCB and FGRF, and refined into a coding framework, which HCE used to systematically code all transcripts. QSR NVivo 2.0 was used to facilitate storage and coding of data.

## Results

Interview and focus group participants were between 55 and 77 years old (mean = 65.2, standard deviation = 6.9). Nineteen were female, all were of 'White British' ethnicity, and there was a wide range in socioeconomic background: the median Carstairs deprivation index was 3. This index is based on postal code classification from 1991 census, with a range from I (most affluent) to VII (most deprived) [19,20]. The current sample included individuals from all seven levels.

### Declining to participate

The explanations given for declining trial participation could be divided into two distinct groups relating to perceived necessity and perceived risk. For one group (N = 6), respondents' explanations indicated a lack of necessity to participate in the trial, and were characterised by mentions of largely mundane problems (either trial-specific or more circumstantial) that nonetheless took priority.

Trial-specific explanations included concerns about the trial involving too much commitment or too long a duration, particularly for individuals at the upper end of the age range.

"I think it was too much of a hassle for me [...] and I thought well if it's not too serious, which might sound stupid, I'm not gonna bother... At the end of the day would it have been beneficial?" (P3.5, male, 58, declined trial)

"The thought of this going on for five years, seemed to me a long time, I didn't want to commit myself." (P3.6, female, 73, declined trial)

A procedural aspect of the trial was the focus of concern for two respondents - the blood tests conducted at the baseline appointment to measure serum cholesterol levels, which led to fear and discomfort in these two, and thus to their declining to participate.

"Well the nurse said I was a fraction over and I could go on the trial. I said I didn't mind that but they was gonna take some blood from me. And I says, 'no way!' That was it. Because I'm terrified of needles" (P3.11, female, 73, declined trial)

Circumstantial explanations emphasised issues such as unfortunate timing due to unrelated health problems at the time of recruitment:

"I was happy to take part in it, but unfortunately I was in [the hospital] having problems and that's why I didn't take part, and I was going to see a specialist and there was a possibility it was either a- well they thought- a hernia, or an ulcer, so I spoke to my own doctor and he said obviously not to take part in this until we'd sorted the other problem out." (P3.2, female, 62, declined trial)

A few reported not seeing any personal benefits or incentives to participate in the trial, with one mentioning lack of financial incentive.

These largely mundane reasons had overridden any initial desire to take part in the trial. The priority of these reasons, and the lack of perceived necessity to participate (for example, "it's not too serious", P3.5), demonstrated the lack of risk perceived from the ankle brachial index result for these respondents.

In contrast, the second group (N = 5) were characterised by a *high *level of risk perceived from participating in the trial. For three respondents this translated into an unwillingness to accept the blinding and random allocation in the trial, and most importantly the possibility of being allocated to the placebo. One respondent disliked the idea of being a 'guinea pig' and acted on this by requesting aspirin on prescription from his own general practitioner, while one respondent chose the less formal route of self-medicating with over-the-counter aspirin.

"I tried to analyse what you were offering under the aspirin trial and when I found out you knew I was at risk, but you were prepared to put me on a dummy pill I said, get lost! And [...] they can't tell you what you were on. So I walked out of the trial at that stage and I went to my own doctor... I spoke to him and told him what was happening, and he said, 'Do you want to go on aspirin?' and I said, 'Well it sounds like I might have to. I'd sooner be on it rather than nothing.' So I have the 75 mg aspirin." (P3.8, male, 68, declined trial)

"I buy [aspirin] in Superdrug, and I just take one you know dissolved in water most days, maybe five times a week" (P3.10, female, 61, declined trial)

For two respondents the risk they perceived from *aspirin *led to their declining. Despite being keen to participate, one man had previously experienced adverse reactions to aspirin, and thus stated that he would have participated if he could have been guaranteed to receive the placebo.

"they had said in the letter it was aspirin...I thought well I'll go and see er...I get tummy trouble, so I try to avoid aspirin if I can [...] and I didn't really fancy going on a prolonged sort of dose of it. So that was the main reason I didn't go on the trial. [...] I said, 'well I don't really want aspirin', [thinking that] they might just give me the placebo, but to some extent the trial's not really working properly if I go in and say, 'well I don't want aspirin but I'll go on the dummy'" (P3.1, male, 59, declined trial)

A second respondent who declined in order to avoid aspirin reported a long history of adverse reactions to various medications and now sought to avoid all conventional medication. For her this avoidance included the placebo:

"I thought, no I'll try and take care of myself other ways [...] I take supplements, I'm also vegetarian anyway, so I thought I would increase my fruit and veg, I take oils, I read a lot of health supplements, I also attend an Ayuverdic doctor. [...] I didn't know what was in the dummy pill! The placebo, I didn't know what was in that, so I wasn't willing to swallow it [either]." (P3.9, female, 60, declined trial)

In comparison to the first group, the risk perceived by this second group was associated with a perceived necessity to engage in action of some sort - seeking aspirin or identifying other lifestyle behaviours with the aim of minimising the risk associated with their ankle brachial index result.

### Stopping trial medication

For the respondents who had started to participate in the trial but had stopped the medication, their explanations for stopping fit into four main categories: Side effects attributed to the trial medication; starting a course of aspirin or medication contraindicated to aspirin; experiencing an outcome event [13]; and 'changed mind'.

Seven respondents described side effects they had experienced and had attributed to the trial medication. They reported consulting the trial team or their own doctor, and being advised to stop the trial medication, either temporarily or permanently. Side effects ranged from knee swelling to tongue tingling, but the most typical were gastro-intestinal as discussed by focus group participants:

FG2.4: again I found that I had stomach problems with the tablet so I assumed that it must be the aspirin (yeah) I was taking it and I kept saying, 'I can't be ill, I don't even know what it is.' I stopped it and started it on two occasions and did find that I was waking up at night with a slight gnawing feeling in the stomach and

FG2.2: I was exactly the same

FG2.4: Did you have the same? Well that's good to know yeah

(FG2.4: female, 63; FG2.2: female, 61)

No respondent expressed anger in relation to these side effects (even when probed in the interview) or reported regret for participating despite the effects being severe in some cases:

"I really felt I'd got gall bladder trouble again because [the pain] was from here right through into me kidneys and really severe. So I went to my GP, and she just checked round and said straight away, 'Don't take anymore, and ring [the trial] and tell them' [...]

HCE: so once you got it sorted out, how did you feel about the trial then?

I felt like well I've let them down, but at same time your health does come first and if that tablet aspirin were gonna kill me...!

HCE: did you feel angry for getting the symptoms that you got?

No, no, because you go into it and you do know what you're doing I mean that were it, it were just one of them things, there's some people as you know who've started with it and they've still no problem." (P2.3, female, 55, stopped medication)

Indeed this respondent's comment about letting the trial down and disappointment in having to cease full participation in the trial was shared by others (for example most of the focus group participants). Furthermore, one respondent's disappointment about having stopped led him to contact the trial with the aim of restarting after a side effect-free period.

Six respondents had been prescribed aspirin, or a medication contraindicated to aspirin, for a range of conditions. Again there was an indication of disappointment in some of these respondents, but rather than an emotional reaction it was typical for the respondent to simply describe what happened in a somewhat resigned manner, accepting the requirements of the changes to their health. For example:

"I took angina, and I went and I [came] off the trial

Husband: and you'd then to start taking aspirin, you didn't know whether you were on aspirin or the placebo under the trial

Aye, and so that's why I come off it, but we're now taking an aspirin a day." (P2.11, female, 76, stopped medication)

"It was the fatal type of malaria I had...I was all wired up the whole time I was in [hospital] and they discovered I had heart fibrillation... After that I'd to go on warfarin you see, so that's why I had to drop out because warfarin and aspirin just don't agree" (P2.5, female, 77, stopped medication)

One respondent had experienced a myocardial infarction eleven months into the trial, which was one of the AAA trial outcome events. Similarly to those who had experienced side effects he did not blame the trial, rather he seemed to be fatalistic about the event.

HE: So how did you feel about that, about going into the trial?

"I was glad in the sense that they found something that maybe, you know. You don't know if you're taking the real aspirin or the dummy aspirin, I felt in a sense that it was helping, you know, till I actually took the heart attack"

(Later in interview):

HE: Did it change how you felt about the trial after having the heart attack?

"In what sense, do you mean whether the trial contributed? I thought maybe the trial to me in a way highlighted it, [that] my heart was bad you know... No I didn't blame anything on the trial, no, I felt that after all if it was gonna do it, then it was gonna happen you know..." (P2.8, male, 63, stopped medication)

In contrast to the medical explanations presented so far, the final category of explanation can be classified as 'changed mind'. Examples of this included two respondents who initially reported stopping the medication due to forgetfulness, but later in their interviews revealed an underlying dislike of medication, as demonstrated in the two quotes from the following respondent at different points in his interview:

"The only reason I stopped was simply because I kept forgetting to take the tablets you know [...] I'm getting terrible forgetful. And I mean it would just be a sort [of] sham for me to say I've been taking it when I haven't, you know. I think trials are- they are good things you know..."

(Later in interview):

"If you're taking a lot, it knocks the hell out of your stomach. [...] Given the choice, I'd rather not take medication full stop." (P2.6 male, 55, stopped medication)

Two respondents explained the lack of necessity they attributed to taking medication associated with the lack of risk they perceived from the ankle brachial index (echoing one group of explanations for declining).

"I think if it had been medication that I needed to take, I would have taken it. And that sounds as though I wasn't committed, but I was when I started certainly, but you know I'd go to take them and 'goodness me, I haven't taken them this week'." (P2.4 male, 72, stopped medication)

These two respondents described managing without medication all their life and thus seeing no reason for starting now.

In this sample, respondents who stopped the medication due to changing their mind seemed to do so early on, whereas most of those who stopped for medical reasons had taken the trial medication for a longer period - up to 3 years in some cases - by the time that they stopped.

## Discussion

The current findings add further insight to the growing literature about trial participation by identifying key reasons for declining to participate, and for stopping medication, in a double-blind placebo-controlled preventive medicine trial. First the accounts of eligible individuals who declined to participate in the trial suggest two distinct groups of explanation: for one group there was a lack of perceived necessity to engage in any action (including participating in the trial) which was associated with a lack of perceived risk from the ankle brachial index, and the explanations focused instead on largely mundane issues. In the second group the central concern was the risk perceived from participating in the trial. The accounts of trial participants who had stopped their trial medication revealed four main categories of explanation - the first three related to medical events (experiencing side effects attributed to the medication, starting a course of medication that would interfere with the trial, and experiencing an outcome event), and a final category which can be summarised as 'changing one's mind'.

The explanations for declining that focused on the risks perceived from the trial - including being blinded, randomised and thus no guaranteed receipt of aspirin - echo a treatment preference found by previous studies [3,5,7]. Our findings revealed both preferences for receiving, and for avoiding the treatment. In previous studies respondents' preferences have been associated with the benefits and drawbacks of an 'experimental' treatment [3,5]. In contrast, our respondents' preferences were associated with personal and public knowledge about aspirin due to its familiarity and widespread use [16] and demonstrated respondents' lack of clinical equipoise. What the respondents overlooked, however, was that the effectiveness of aspirin as a primary prophylactic was still unknown and the purpose of conducting the AAA trial. Indeed the recently published AAA trial results demonstrate that these respondents' strong preference for aspirin was not supported [13].

The findings demonstrate the importance of 'mundane' explanations for declining which were not associated with discontent with randomisation or treatment allocation, but which focused on procedural aspects of the trial or unrelated health circumstances at the time of invitation. For these respondents participating in the trial was not perceived as sufficiently attractive or necessary to override these factors. Indeed these accounts revealed a lack of perceived necessity for *any *action to address the increased risk (including participating in the trial), which highlights the lack of risk that these respondents perceived from their ankle brachial index result and/or atherosclerosis.

While there is a clear distinction for trialists between individuals who decline to participate and trial participants who stop their trial medication, the current data demonstrate some overlap in the explanations given by the two groups: those who stopped the medication because they 'changed their mind', and those who declined to participate for 'mundane' reasons shared the same lack of necessity for action and a lack of perceived risk from the ankle brachial index.

That medical reasons (both related and unrelated to the trial) result in some participants stopping the trial medication is inevitable in a trial of long duration with individuals of older age and/or increased risk. Outcome events are to be expected, as analysing the proportion of participants in each arm who experience an outcome event is after all the main purpose of a trial. Furthermore establishing the number and type of side effects experienced is another crucial element of the trial's analysis. Of interest, however, was the disappointment expressed by respondents who had experienced an outcome event or side effect and to some extent by those whose medical circumstances had required a prescription of aspirin or other medication thus preventing them continuing to take the trial medication. Such discontent was not directed at the trial for possibly having caused the side effect or not having prevented the outcome event, rather it emerged as regret at having to end their full participation in the trial accompanied by a feeling that they had let the trial down. This is in line with previous findings that a commonly reported driver for participating in research is the desire to help others [4,7,11], and that the participatory experience can lead to the feeling of a 'warm glow' [21]. Many of these respondents appeared to accept this change in circumstances (and thus the requirement to stop the trial medication) in a somewhat fatalistic and resigned manner, and pointed out that when the risks to their health outweighed the benefits of participating, it is understandably their health that takes priority.

### Strengths and limitations

The current paper extends our knowledge about trial participation by exploring the perspectives of those invited to participate in a previously unexplored context - a double-blind placebo-controlled randomised trial in preventive medicine with a community population. The findings will be relevant to other clinical trials in particular preventive medicine and community based trials. Some of the findings are likely to be specific to the use of aspirin - for example the strong treatment preferences expressed by some respondents which were associated with their own personal knowledge and also public awareness about aspirin [16] - although these findings will be useful to trials with other 'commonplace' medicines.

Sampling in this study was subject to the usual response bias of interview studies with an opt-in recruitment process. The sample of participants who had stopped the trial medication was limited to those who were happy to be followed up by the trial team. However the resulting sample comprises two groups of individuals that can be difficult to access; in particular the accounts of participants who have stopped trial medication are under researched.

### Implications

The findings will be of interest to those running trials (particularly in a community population and/or preventive medicine) to address factors that may be affecting recruitment and adherence, while ensuring that conduct remains ethical. Asking individuals for consent to be contacted again at a later date could prove an effective strategy for those who are keen to participate in the trial but decline due to health problems not related to the trial or other life circumstances. This may be useful in trials with long recruitment periods. For individuals who have concerns about particular procedural aspects of a trial, procedures could be put in place to encourage potential recruits to engage in discussion with a research nurse allowing them to express their concerns and explore possible solutions, and even making minor protocol adjustments in special cases.

The finding that a substantial group of our respondents did not regard participation (or indeed any action) as necessary demonstrated the little risk perceived by these respondents from their ankle brachial index result. This highlights the importance of ensuring that potential recruits understand the meaning of the results of any clinical measures used which make them eligible for a trial.

With individuals who see no necessity to participate, the potential benefits to others and to medical research that may result from the trial could be emphasised [22] though equal time should be spent discussing all options and ensuring that coercion is avoided [9].

For potential recruits with strong treatment preferences it would be unethical to attempt to persuade them to participate in a blind placebo-controlled trial. Furthermore when the treatment is widely available as an over-the-counter medication, like aspirin, such individuals will seek the medication by themselves as demonstrated by a couple of our respondents. However, where feasible a patient preference trial design would enable inclusion of such participants.

The inevitable occurrence of side effects and health events in a trial population such as the AAA population highlights the importance of ensuring that potential recruits are provided with understandable written information about likely side effects and procedures to follow in the event of experiencing them, and contact details of trial staff that can help with any query (as was the case in the AAA trial). Informing potential recruits about all the possible side effects may lead to slower recruitment by putting some individuals off, but the resulting sample may be one that adheres for a longer duration. Recruitment of trial participants is expensive so any efforts that can result in a longer lasting sample are worth investing time and effort into.

## Conclusions

The reasons for declining to participate in randomised controlled trials and the reasons for trial participants stopping their trial treatment are of key importance to clinical researchers who are concerned with optimising both recruitment and adherence, while ensuring conduct remains ethical and non-coercive. With regards to the AAA trial we have outlined two distinct categories of reasons for declining: strong treatment preference (high perceived risk from the trial and/or the ABI result) and lack of perceived necessity (low perceived risk from the ABI result). The level of risk that a respondent perceived (from their ABI result and its meaning, from randomisation, from taking aspirin, or from not taking aspirin) and the level of perceived necessity to engage in action of some sort (including participating in the trial) were the key drivers. The categories of reasons for stopping medication fit into two distinct groups: changing one's mind (associated with a low perceived risk from the ABI result and lack of perceived necessity - echoing one of the declining categories), and medical reasons (which are inevitable in a trial involving people of older age and/or increased risk).

Adjustments to trials could address these issues of declining and stopping; where feasible patient-preference arms may be useful for addressing strong treatment preferences, minor protocol adjustments could be made for individuals with concerns about procedural aspects, more time could be spent with potential recruits ensuring comprehension of eligibility criteria (in this case the meaning of a low ABI) and discussing the risks and benefits of the trial, and for individuals who decline due to temporary circumstances (such as unrelated health issues), consent could be sought to contact them at a later date.

These findings indicate that in planning future trials (especially in preventive medicine) particular attention is given to designing appropriate recruitment materials and processes that fully inform potential recruits of the risks and benefits of participation, and incorporate time for discussion of the issues that our respondents raised.

## Competing interests

HCE and SCB declare that they have no competing interests.

FGRF is Principal Investigator, JFP is Co-Investigator, and MCWS coordinated the AAA trial.

## Authors' contributions

HCE undertook the research as part of her doctoral thesis under supervision of SCB and FGRF. The idea for this paper was conceived by HCE, MCWS and JFP. HCE wrote the first and all subsequent drafts of the paper, with input from the other authors. All authors read and approved the final manuscript.

## Appendix

### Relevant Section of Topic guide

Individuals who declined to participate

• Reasons for declining to participate in the trial

• Perceptions of why people choose to participate

• Views on not knowing the trial tablet's identity

Participants who stopped medication

• Experience of taking part in the 'aspirin trial'

• Reasons for participating in the trial

• Feelings about taking the trial tablet for 5 years

• Feelings about not knowing the tablet's identity

• Reasons for stopping the trial medication and subsequent reflections

All respondents

• Advantages and disadvantage of taking part in the trial

• Prior trial experience
